# Report of Deaths in the City of Buffalo for the Month of November, 1861

**Published:** 1862-01

**Authors:** J. Whitaker

**Affiliations:** Health Physician


*Report of Deaths in the City of Buffalo for the month of November,*1861.

Accident 8, Albuminuria 1, Angina 1, Apoplexy 1, Bronchitis 1, Cancer 1, Cancer of the Womb 1, Consumption 19, Convulsions 9, Croup 5, Debility 6, Delirium Tremens 4, Dentition 1, Diarrhœa 2, Disease of the Brain 2, Disease of the Heart 3, Disease of the Lungs 2, Disease of the Liver 1, Diphtheria 3, Dropsy of the Brain 5, Dysentery 1, Empyema 1, Fever 2, Inflammation of the Brain 2, Inflammation of the Lungs 5, Inflammation of the Lungs Typhoid 2, Inflammation of the Peritoneum 1, Inflammation of the Stomach 1, Inflammation of the Veins 1, Intemperance 2, Marasmus 5, Measles 1, Old Age 6, Paralysis 1, Scrofula 1, Scarlet Fever 11, Softening of the Brain 1, Suicide 1, Syphilis 1, Typhoid Fever 2, Typhus Fever 1, Whooping Cough 3, Unknown 4, Still Born 7. Total 143. Under one year of age 34. Between one and twenty 52. Between twenty and fifty 34. Over fifty 23. Males 79; Females 63; not given 1; Total 143.

J. WHITAKER, Health Physician.

